# Vegan Diet in Dermatology: A Review

**DOI:** 10.3390/jcm12185800

**Published:** 2023-09-06

**Authors:** Hannah Lee, Nathan Sim, Audrey Fotouhi, Steven Daveluy

**Affiliations:** 1 School of Medicine, Wayne State University, Detroit, MI 48201, USA; hannah.lee@med.wayne.edu (H.L.); nathan.sim@med.wayne.edu (N.S.); 2 Department of Dermatology, Wayne State University, Detroit, MI 48201, USA; sdaveluy@med.wayne.edu

**Keywords:** vegan diet, nutrition, skin, kwashiorkor, inflammation, dermatitis, acne, psoriasis, hidradenitis suppurativa, atopic dermatitis, eczema, gut–skin axis, immunity

## Abstract

Diet is known to play a role in the development of skin disorders. While a vegan diet is frequently described as a risk factor for skin disease secondary to nutritional deficiency, this risk may be overestimated. This review aims to debunk myths and provide information on skin disorders and inflammatory skin conditions that have associations with specific nutritional deficiencies in the context of a vegan diet. A literature search was performed for each nutrient and inflammatory skin disease using the PubMed/MEDLINE database and public health website pages concerning a vegan diet. The literature has individual cases reporting skin disease due to deficiencies in vitamin B2 and vitamin A in patients following a vegan diet. The recommended daily amounts of nutrients and vitamins can be fulfilled on a vegan diet. Vegan diets also avoid food groups such as dairy and other animal-based products, which holds benefits in inflammatory skin diseases including acne, psoriasis, hidradenitis suppurativa, and atopic dermatitis. Overall, the risk of skin disease secondary to nutritional deficiency in patients following a vegan diet is very low and likely over-emphasized. A well-balanced and conscientiously planned vegan diet can adequately provide the necessary amounts of proteins, vitamins, and minerals to support skin health.

## 1. Introduction

The role of diet in maintaining skin health cannot be overstated. Both inadequate and excess nutrient consumption may be associated with skin disorders, including acne vulgaris, psoriasis, hidradenitis suppurativa, and atopic dermatitis [1]. Diet is a main contributor to diversity in the gut microbiome. An altered gut microbiome, or gut dysbiosis, is associated with an altered immune response, promoting the development of such skin diseases [2].

Despite the importance of nutrition in skin health, there is much confusion surrounding the ideal diet for optimal skin health, particularly for those following a vegan diet. A vegan diet, which excludes all animal-derived products including meat, poultry, seafood, eggs, dairy, gelatin, and honey, may increase the risk of certain nutritional deficiencies, some of which are associated with skin disorders. The vegan diet includes the consumption of plant-based foods such as nuts, grains, seeds, legumes and derivatives, fruits, and vegetables. Nevertheless, ensuring a sufficient intake of vitamins and nutrients through a healthy vegan diet and appropriate supplements can prevent nutritional deficiencies.

In a recent comprehensive review of nutritional deficiency and skin disease, a vegan diet was listed as a risk factor for riboflavin and vitamin A deficiencies, and a potential risk factor for lower protein intake [3,4,5]. A vegan diet was the only diet highlighted as a risk factor for deficiency; thus, other diets were not included as a focus of this review [2,3]. In addition, nutritional deficiencies associated with veganism that do not cause frequent dermatologic manifestations are outside the scope of this review. For example, vitamin B12 is only present in animal products, and thus, a vegan diet lacks vitamin B12 completely and supplementation is required [6]. Vitamin B12 was not included in this review as the manifestations of deficiency are rarely cutaneous.

This review will discuss nutritional deficiencies that cause dermatologic manifestations, including both clinical signs and prevention, for which a vegan diet is a risk factor. This review will also discuss the impact of a vegan diet on several common inflammatory skin diseases.

## 2. Materials and Methods

For the literature search in the PubMed/MEDLINE database, the search terms included “vegan diet”, “veganism”, “plant-based diet”, “vitamin B2”, “riboflavin”, “riboflavin deficiency”, “vitamin A”, “vitamin A deficiency”, “Kwashiorkor”, “true protein deficiency”, “nutritional deficiency”, “dairy”, “acne”, “psoriasis”, “hidradenitis suppurativa”, “atopic dermatitis”, “inflammatory skin disease”, and “diet gut microbiome skin health”. This search was supplemented with additional information on the vegan diet and recommendations for adequate nutritional intake and Harvard University School of Public Health articles. Only published articles that were published in English were selected. After titles and abstracts were screened for relevance and the results were thoroughly examined, 74 articles were selected to be included in this narrative review.

## 3. Results and Discussion

### 3.1. Nutritional Deficiencies

#### 3.1.1. Riboflavin (B2)

Riboflavin, or vitamin B2, is a water-soluble vitamin involved in a variety of reduction–oxidation reactions. Its active forms, flavin adenine di-nucleotide (FAD) and flavin mononucleotide (FMN), act as electron carriers in the respiratory electron transfer chain, and FAD is necessary for the oxidation of fatty acids [7].

Riboflavin deficiency classically presents with angular cheilitis and glossitis, occasionally with a confluent erythematous, scaly dermatitis of the scrotum or vulva and thighs, with sparing of the midline, as the initial cutaneous manifestation [4]. During embryonic development, riboflavin deficiency results in muscular, skeletal, and gastrointestinal abnormalities [8]. Extracutaneous manifestations in adults include anemia, decreased iron absorption, neurodegeneration, and peripheral neuropathy [7].

Although some studies have shown lower intakes of riboflavin in a vegan diet compared to a non-vegan diet, clinical riboflavin deficiency has not been reported in adults [9]. A case study reported a male neonate presenting with life-threatening hypoglycemia and lactic acidosis due to maternal riboflavin deficiency. The mother followed a strict vegan diet with occasional supplementation of vitamin B12, folic acid, vitamin D, and omega-3. This case highlights the importance of riboflavin intake for pregnant women on a vegan diet [10].

The recommended dietary allowance (RDA) for men and women over the age of 19 is 1.3 mg and 1.1 mg, respectively, with higher RDA levels for pregnant women [11]. Plant-based foods that provide about 1 mg per serving of riboflavin include asparagus, bananas, beans, broccoli, figs, kale, lentils, peas, seeds, sesame tahini, sweet potatoes, tofu, tempeh, wheat germ, and enriched bread [9]. Pregnant women on a vegan diet may need additional supplementation.

#### 3.1.2. Vitamin A

Vitamin A is a fat-soluble isoprene derivative with several forms including retinol, retinoic acid, and beta-carotene. Vitamin A plays an important role in the differentiation of epithelial tissues, protein synthesis in the eye, and modulation of the immune system [12,13,14]. Overall, vitamin A deficiency is relatively rare. It can lead to abnormal epithelial keratinization, manifesting clinically as phrynoderma, generalized xerosis, and hair casts [3]. Classic ophthalmologic manifestations usually co-exist with cutaneous disease and include xeropthalmia and night blindness [8].

One case reports a child developing keratomalacia while on a strict vegan diet [15]. He refused to eat most fruits, vegetables, and cooked meals, and his diet consisted only of non-fortified soy milk, potato chips, rice cereal, and juice. The authors suggest that a vegan diet puts children at risk for anemia, osteopenia, and protein and zinc deficiencies. However, this child’s extremely restrictive diet should not be generalized to all vegan diets. The Academy of Nutrition and Dietetics states that “appropriately planned vegetarian, including vegan, diets are healthful, nutritionally adequate, and may provide health benefits for the prevention and treatment of certain diseases”. They also state that vegan and vegetarian diets are appropriate for all stages of life, including pregnancy, lactation, infancy, childhood, adolescence, and older adulthood, and for athletes [16].

A well-balanced vegan diet contains ample vitamin A, although a restrictive or bizarre diet (which may happen to be plant-based), could certainly lead to vitamin A deficiency. Plant-based sources of vitamin A include leafy green vegetables, orange and yellow vegetables, tomatoes, red bell pepper, cantaloupe, and mango, as well as fortified breakfast cereals, breads, and juices.

#### 3.1.3. Kwashiorkor

In the public, a common concern about veganism is protein deficiency. True protein deficiency leads to kwashiorkor, which should not be confused with marasmus, a form of nutritional deficiency caused by a lack of total calories. Kwashiorkor is exceeding rare in developed countries such as the United States [17]. It has rarely been described in developed countries, occurring in infants who were fed rice milk instead of breast milk or formula [18]. Kwashiorkor can classically present with the cutaneous manifestation of “peeling enamel paint”, starting at sites of friction and progressively generalizing as the disease advances [19]. Extracutaneous manifestations include gross edema and abdominal distention.

To prevent protein deficiency, the National Academy of Medicine recommends that adults consume a minimum of 0.8 g of protein per kilogram of body weight per day. A vegan diet can easily support the recommended daily protein intake as protein is found in nearly all plant foods, including legumes, nuts, seeds, and whole grains [20]. A review article demonstrates that there is no evidence of protein deficiency among individuals on a plant-based diet in Western countries [5].

Traditionally, proteins were categorized as “complete”, meaning they contain all essential amino acids, or “incomplete”, meaning they lack one or more of the nine essential amino acids [20]. Animal-derived foods (meat, poultry, fish, eggs, and dairy foods) tend to be good sources of complete protein, while individual plant-based foods (fruits, vegetables, grains, nuts, and seeds) often lack one or more essential amino acids [21]. Those following a vegan diet who eat a variety of plant-based foods daily have no issue consuming all of the essential amino acids necessary for function. “Complete” plant proteins exist, and examples include quinoa and chia seeds [21]. Table 1 summarizes nutritional deficiencies associated with a vegan diet.

### 3.2. Inflammatory Skin Disease

#### 3.2.1. Acne Vulgaris

Acne vulgaris is an inflammatory skin disorder that involves excess sebum production induced by androgenic hormones and insulin-like growth factor 1 (IGF-1) in hair follicles [22]. Though the exact pathogenesis of acne triggers remains unclear, genetic, hormonal, lifestyle, and environmental factors have been shown to affect the development of acne [22]. Historically, the relationship between diet and acne has been highly controversial.

The consumption of dairy products such as cows’ milk has been correlated with acne. This may be explained by a positive association between increased milk consumption and IGF-1 concentrations [23,24]. For adolescent boys, higher levels of skim milk consumption were associated with a higher prevalence of acne (*p* = 0.02), while for adolescent girls, significant associations were present with whole milk, low-fat milk, and skim milk (*p* < 0.005) [22,23]. Another study of female nurses also found a positive association between skim milk intake and acne (*p* < 0.005) [25].

Foods with a high glycemic index (GI) have also been posited to worsen acne [26]. In a randomized, controlled trial comparing a low- versus high-glycemic-load diet, after 12 weeks, the low-GI group had a statistically significant reduction in acne lesion counts compared with the high-GI group (*p* = 0.01). The low-GI group also had reduced weight, reduced free androgen levels (*p* = 0.04), and increased IGFBP-1 levels (*p* = 0.001) [26]. As an additional finding, an increase in the ratio of saturated to monounsaturated fatty acids of skin surface triglycerides was observed in the low-GI group, as well as an increase in follicular sebum outflow, which was associated with an increase in the proportion of monounsaturated fatty acids in sebum [27]. Although the exact mechanisms by which the gut microbiome can influence the development of acne are unknown, a diet high in fat or foods with a high GI affects the intestinal microbiota by increasing intestinal permeability, which may aggravate the development of acne [28]. Therefore, modulation of the gut microbiome or gut dysbiosis could potentially influence the appearance and evolution of acne [28]. These findings suggest that GI, among other dietary components, may play a role in the pathogenesis of acne.

Other studies support the positive correlation between high-GI foods and acne. For example, a decrease in sebaceous gland size and sterol regulatory element-binding protein-1 expression in facial acne occurred after 10 weeks of a low glycemic load diet in 17 patients [29]. The subjects in the low glycemic group also demonstrated significant clinical improvement in the number of acne lesions. In addition, a systematic review found that high GI and increased daily glycemic load intake were positively associated with acnegenesis and acne severity [30]. Overall, the consumption of certain dairy products, such as cows’ milk, and foods with a high GI has a proacnegenic effect.

Dairy avoidance and a low-GI diet could be advantageous for patients with acne. Veganism, being a diet devoid of animal-based products and, when well-balanced, high in low-GI foods like fruits and vegetables, may promote anti-acnegenic effects. In fact, dietary intake of soy-based products appears to lower the incidence of acne [31]. This effect may be attributable to the isoflavones and phytoestrogens in soy, which oppose androgen-induced sebum production [32]. When 160 mg/day of soybean isoflavones was given to patients with acne for 12 weeks, there was a significant decrease in the number of acne lesions, likely due to decreased dihydrotestosterone levels [33]. Soy protein may reduce visceral body fat, which could also improve acne [34]. In general, the consumption of fruit and vegetables, which are foods with a low GI, may play a protective role due to their anti-inflammatory effect. Therefore, a well-balanced vegan diet may be beneficial in preventing and reducing acne lesions.

#### 3.2.2. Psoriasis

Psoriasis is a Th1-driven chronic inflammatory skin disease with characteristic proinflammatory cytokines. Psoriasis is often comorbid with metabolic syndrome (MetS). MetS refers to the co-occurrence of several cardiovascular risk factors, including insulin resistance or type 2 diabetes, obesity, atherogenic dyslipidemia, and hypertension [35,36]. The levels of proinflammatory cytokines are elevated in psoriasis, as well as in obesity and ischemic heart disease, and these cytokines have been shown to influence fat deposition, insulin action, and lipid metabolism [36]. Thus, chronic inflammation may be the link between psoriasis and MetS, and the appropriate treatment of MetS could be crucial in the management of patients with psoriasis.

Diet is a relevant environmental factor in psoriasis since malnutrition, inadequate body weight, and metabolic disease may make the psoriasis more treatment-refractory [37]. A cross-sectional study examining food intake patterns in psoriasis patients found two main dietary patterns. Pattern 1 was predominantly processed foods, while Pattern 2 was predominantly fresh foods. Adherence to Pattern 2 was associated with normal serum lipids and blood pressure, a lower waist-to-hip ratio, and decreased psoriasis (skin) activity (*p* = 0.001) [37]. A national survey to identify common dietary habits, interventions, and skin outcomes in response to the interventions among patients with psoriasis found that the percentage of patients reporting skin improvement was highest after reducing alcohol, gluten, and nightshades and after adding fish oil/omega-3, vegetables, and vitamin D. Moreover, most patients reporting a favorable skin response followed Pagano, vegan, and Paleolithic diets [38]. The Pagano diet includes an increased intake of fruits and vegetables and a decreased intake of nightshades and processed foods. As diet affects the gut microbiota, and the gut microbiome interacts with the skin system via the immune system (IL-23/IL-17 signaling pathways), the regulation of gut dysbiosis through a healthy diet may play an important role in the improvement of psoriasis and skin inflammation [39]. The aforementioned studies demonstrate that diet plays a role in managing psoriasis and its associated comorbidities.

A plant-based diet may have beneficial effects on psoriasis, as the elimination of animal-based products limits the intake of saturated and trans fatty acids, improving skin and preventing comorbidities associated with psoriasis [32,40]. In fact, it is not possible to consume cholesterol in a vegan diet as cholesterol is derived from animals. Replacing meat with an increased intake of vegetables, fruits, legumes, nuts, and cereal products yields a diet low in saturated and trans-fat and arachidonic acid, and high in antioxidants and omega-3 fatty acids [36,41]. Omega-3 fatty acids have anti-inflammatory properties compared to omega-6 fatty acid arachidonic acid, which is a precursor to a number of potent pro-inflammatory mediators [42,43]. Soy is a legume that may protect against psoriasis as the isoflavones in soy, particularly genistein, have anti-inflammatory activity [44,45]. Moreover, a plant-based diet results in increased potassium intake which may cause an increase in the synthesis of cortisol, having an anti-inflammatory effect [46]. On top of improving psoriasis skin manifestations, plant-based diets such as the vegan diet have been adopted to improve body weight and composition, as well as MetS [47,48].

#### 3.2.3. Hidradenitis Suppurativa

Hidradenitis suppurativa (HS) is another chronic inflammatory disorder characterized by chronic deep-seated nodules, abscesses, and scars [49]. Though the exact mechanism of HS remains unknown, it is hypothesized that inflammatory cytokines and androgenic hormones play a role in the pathogenesis of HS [50,51]. HS is associated with many comorbidities: hypertension, obesity, dyslipidemia, thyroid disorder, arthropathies, psychiatric disorders, and polycystic ovarian syndrome, most of which are inflammatory in nature, with the most common comorbidity being obesity [50]. The management of HS requires the control of active inflammation and subsequent surgical management to treat scarring. Though many new promising treatments are emerging for HS, the use of dietary modification to manage disease activity has been of considerable interest, yet the relationship between diet and HS remains controversial [49].

Although it is unclear whether diet may prove to significantly limit the severity of HS, some studies suggest that brewer’s yeast exclusion diets, weight loss through dietary interventions, and bariatric surgery each have the potential to improve HS symptoms [49,52,53,54,55,56]. Examples of dietary interventions that have been reported to be helpful for some patients include eliminating dairy products, limiting simple carbohydrate and sugar intake, and avoiding nightshades [49]. An online survey of HS support groups identified alleviating and exacerbating foods in HS severity/flares [57]. Some reported that vegan HS-alleviating foods include fruits and vegetables, while sweets, carbohydrates (bread, pasta, rice), dairy products, and high-fat foods were found to be exacerbating. According to preliminary observations, a healthy dairy-free and low-glycemic-load diet may provide relief from the progression of HS lesions and possibly prevent new lesions, as the initial obstruction of the follicular duct appears to be mediated by the hormonal influences of dairy products and foods with a high GI [51,58]. Specifically, a dairy-free diet led to an improvement in 83% of 47 patients with HS, and none worsened. Thus, the avoidance of dairy and foods with a high GI that trigger androgen-induced obstruction of the follicular duct may help to control HS [51].

Currently, there is no existing literature exploring the relationship between veganism and HS. However, bacteria in the fecal samples of HS patients support the possibility of a role for gut microbial alterations in HS [59]. Given the potential benefits of a diet that avoids dairy and foods with a high GI, a plant-based diet such as a vegan diet may be beneficial in the relief and prevention of HS symptoms. Future research may seek to investigate the association between a vegan diet and HS more thoroughly.

#### 3.2.4. Atopic Dermatitis

Atopic dermatitis (AD) is a Th2-driven chronic inflammatory disorder, commonly associated with other atopic manifestations such as food allergy, allergic rhinitis, and asthma [60,61]. Although the role of diet in AD has not been completely elucidated, dietary exposure and the gut microbiome are thought to play a role in the pathogenesis of AD [62,63]. Breast milk has generated significant interest as a means to prevent AD in children due to its immunologic activity [62]. Though some study findings appear to be contradictory, current data suggest that exclusively breastfeeding for three to four months may yield a potential protective effect against AD in children [62,64,65,66].

A diet rich in fruits and vegetables may have a beneficial effect on AD as high amounts of flavonoids are present in fruits and vegetables [62]. Flavonoids are thought to have significant antioxidant and anti-inflammatory effects, which may improve AD symptoms [62]. Interestingly, AD prevalence is higher in children and adolescents consuming fast food and meat burgers [67,68]. This may be because fast food consumption is associated with poor diet quality, high caloric intake, and obesity in children and adolescents; obesity is an independent risk factor for asthma and allergic sensitization, as well [67,69]. Furthermore, dietary fatty acids are known to modulate immune reactions; therefore, it is plausible that dietary fat intake from fast food may play a role in the pathophysiology of atopic diseases [70,71]. A diet rich in fat and low in fiber can alter the gut microbiome, which may yield an inflammatory response as in AD by reducing the production of anti-inflammatory metabolites, specifically short-chain fatty acids [72]. Since the gut and skin have many similar characteristics and communicate with one another, targeting the gut microbiome may be helpful in regulating the immune response and improving AD lesions [72]. A plant-based diet may be an effective way to promote a diverse ecosystem of beneficial microbes that support overall gut and skin health [73,74].

As AD is especially prevalent among infants and children, safe and natural therapeutic options have considerable appeal. In accordance with the aforementioned studies, increasing fruits and vegetables and decreasing the consumption of fast food and meat may prevent and improve AD symptoms. These dietary modifications are supported by a well-balanced vegan diet. However, it should be noted that children should not be put on a restrictive diet to manage AD as there is little evidence to support any beneficial effects of dietary restrictions with potentially harmful effects [62]. Table 2 summarizes the impact of a vegan diet on common inflammatory skin diseases.

## 4. Conclusions

In conclusion, although there are limited data to analyze the relationship between a vegan diet and skin diseases, evidence supports the concept that a well-balanced, whole foods vegan diet can be beneficial for inflammatory skin disease and its associated comorbidities. The existing literature also demonstrates that a well-balanced vegan diet can easily support the recommended amount of daily protein, vitamin A, and vitamin B2. Future research may include more extensive randomized studies regarding the implications of a vegan diet and skin disorders in relation to the gut microbiome.

## Figures and Tables

**Table 1:** Nutritional deficiencies associated with a vegan diet.

Nutritional Deficiency	Associated Dermatologic Conditions	Dietary Sources in the Vegan Diet
Riboflavin (B2)	Angular cheilitis Glossitis	Asparagus, bananas, beans, broccoli, kale, lentils, peas, seeds, sweet potatoes, tofu [10]
Vitamin A	Phrynoderma Xerosis Hair casts	Leafy green vegetables, orange and yellow vegetables, tomatoes, red bell pepper, cantaloupe, mango
Kwashiorkor	Dermatosis described as “peeling enamel paint”	Legumes, nuts, seeds, whole grains [20] Quinoa, chia seeds [21]

**Table 2:** Inflammatory skin diseases and benefits from aspects of the vegan diet.

Inflammatory Skin Disease	Vegan Diet	Associated Benefits
Acne vulgaris	Dairy avoidance Low-GI diet	Anti-inflammatory effect and reduced acne lesion counts [27,28,29,30]
Psoriasis	Plant-based diet No animal-based products	Anti-inflammatory effect Skin improvement and prevention of comorbidities [32,40]
Hidradenitis suppurativa	Dairy avoidance Low-GI diet	Possible prevention of new lesions or progression of existing lesions [51,58]
Atopic dermatitis	Fruits and vegetables Fast food avoidance	Antioxidant and anti-inflammatory effects [62,72]

## Data Availability

No new data were created or analyzed in this study. Data sharing is not applicable to this article.
